# Optimal Targeted Temperature Management for Patients with Post-Cardiac Arrest Syndrome

**DOI:** 10.3390/medicina60101575

**Published:** 2024-09-25

**Authors:** Tsukasa Yagi, Eizo Tachibana, Wataru Atsumi, Keiichiro Kuronuma, Kazuki Iso, Satoshi Hayashida, Shonosuke Sugai, Yusuke Sasa, Yoshikuni Shoji, Satoshi Kunimoto, Shigemasa Tani, Naoya Matsumoto, Yasuo Okumura

**Affiliations:** 1 Department of Cardiology, Kawaguchi Municipal Medical Center, Kawaguchi 333-0833, Japan; 2 Department of Cardiology, Nihon University Hospital, Tokyo 101-8309, Japan; 3 Division of Cardiology, Department of Medicine, Nihon University School of Medicine, Tokyo 173-8610, Japan

**Keywords:** resuscitation, targeted temperature management, therapeutic hypothermia, post-cardiac arrest syndrome

## Abstract

*Background*: To prevent hypoxic–ischemic brain damage in patients with post-cardiac arrest syndrome (PCAS), international guidelines have emphasized performing targeted temperature management (TTM). However, the most optimal targeted core temperature and cooling duration reached no consensus to date. This study aimed to clarify the optimal targeted core temperature and cooling duration, selected according to the time interval from collapse to return of spontaneous circulation (ROSC) in patients with PCAS due to cardiac etiology. *Methods*: Between 2014 and 2020, the targeted core temperature was 34 °C or 35 °C, and the cooling duration was 24 h. If the time interval from collapse to ROSC was within 20 min, we performed the 35 °C targeted core temperature (Group A), and, if not, we performed the 34 °C targeted core temperature (Group B). Between 2009 and 2013, the targeted core temperature was 34 °C, and the cooling duration was 24 or 48 h. If the interval was within 20 min, we performed the 24 h cooling duration (Group C), and, if not, we performed the 48 h cooling duration (Group D). *Results*: The favorable neurological outcome rates at 30 days following cardiac arrest were 45.7% and 45.5% in Groups A + B and C + D, respectively (*p* = 0.977). In patients with ROSC within 20 min, the favorable neurological outcome rates at 30 days following cardiac arrest were 75.6% and 86.4% in Groups A and C, respectively (*p* = 0.315). In patients with ROSC ≥ 21 min, the favorable neurological outcome rates at 30 days following cardiac arrest were 29.3% and 18.2% in Groups B and D, respectively (*p* = 0.233). *Conclusions*: Selecting the optimal target core temperature and the cooling duration for TTM, according to the time interval from collapse to ROSC, may be helpful in patients with PCAS due to cardiac etiology.

## 1. Introduction

Cardiac arrest is a significant public health problem worldwide. The neurological intact for cardiac arrest remains low despite decades of efforts to promote cardiopulmonary resuscitation (CPR) science and education [1]. To prevent hypoxic–ischemic brain damage in patients who remain sedated or comatose following cardiac arrest, international guidelines have highlighted performing targeted temperature management (TTM) [2,3]. In 2002, two studies involving patients who remained unconscious after the return of spontaneous circulation (ROSC) from shockable cardiac arrest due to cardiac etiology compared therapeutic hypothermia with standard treatment [4,5]. A significant improvement in neurologic function with therapeutic hypothermia was observed in these studies. Moreover, the TTM and TTM2 trials in 2013 and 2021, respectively, showed that patients with post-cardiac arrest syndrome (PCAS) who were treated with hypothermia did not have lower mortality than those who were treated with normothermia [6,7]. The HYPERION trial by Lascarrou et al. showed that, in patients with nonshockable cardiac arrest, therapeutic hypothermia at 33 °C led to better neurological outcomes than targeted normothermia [8]. One randomized control study by Kirkegaard et al. showed that TTM for 48 h did not significantly improve 6-month neurologic outcomes compared with TTM for 24 h [9].

Several studies have shown the optimal targeted core temperature and cooling duration as TTM in patients with PCAS [7,8,9,10]. However, to date, the most optimal targeted core temperature and cooling duration reached no consensus [3]. Moreover, various results have been reported about the relationships between favorable neurological outcomes and the target core temperature and cooling duration in the real world [11,12,13,14]. This study aimed to clarify the optimal targeted core temperature and cooling duration, selected according to the time interval from collapse to ROSC in patients with PCAS due to cardiac etiology. In 2000, the National Association of Emergency Medical Service (EMS) Physicians Standards and Clinical Practice Committee suggested that EMS responder resuscitation efforts could be terminated in cardiac arrest patients who do not respond to from 20 to 30 min of CPR [15]. Therefore, in this study, 20 min was used as the cutoff value for the time interval from collapse to ROSC.

## 2. Materials and Methods

### 2.1. Study Design

This was a retrospective single-center study of therapeutic hypothermia in patients with PCAS due to cardiac etiology. The ethics committee of Kawaguchi Municipal Medical Center approved this study (Institutional Review Board number: 2021-11, 28 June 2021). Informed consent was obtained as an opt-out, which was posted at Kawaguchi Municipal Medical Center. Patients who underwent therapeutic hypothermia as post-cardiac arrest care were registered between January 2009 and December 2020. The data, analytic methods, and study materials will not be made available to other researchers to reproduce the results or replicate the procedure.

**Figure 1:**
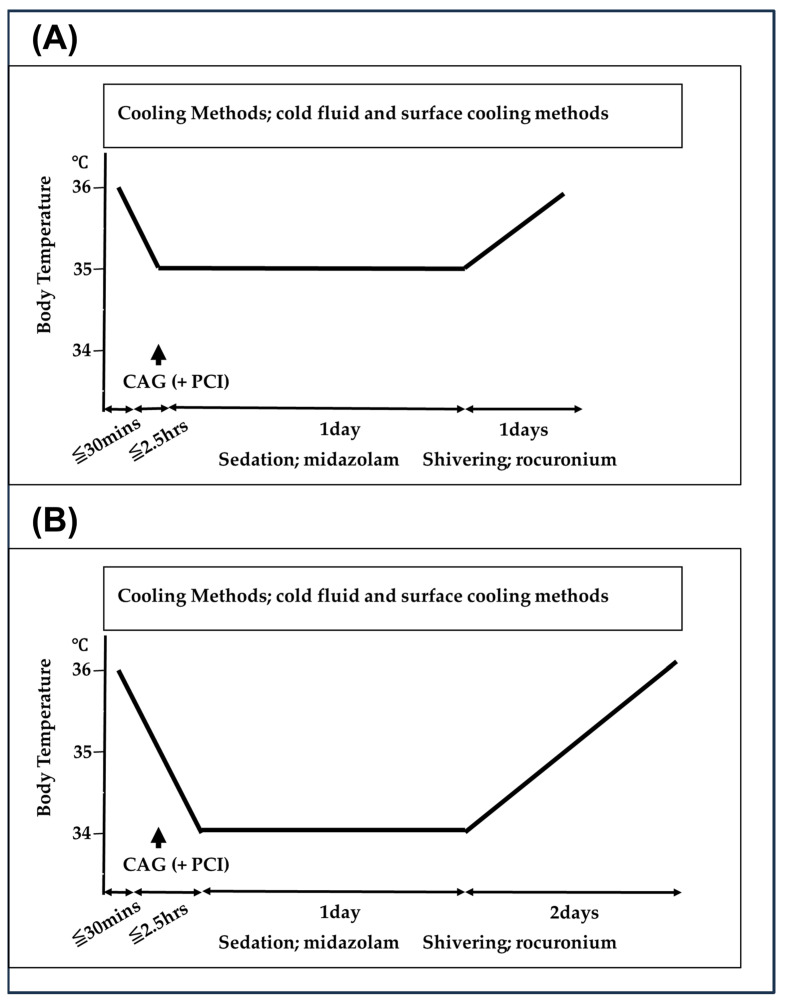
Therapeutic hypothermia protocols from January 2014 to December 2020. CAG indicates coronary angiography; PCI, percutaneous coronary intervention. (**A**) When the time interval from collapse to the return of spontaneous circulation (ROSC) was within 20 min, we performed the 35 °C targeted core temperature, and the cooling duration was 24 h (Group A). (**B**) When the interval was not within 20 min, we performed the 34 °C targeted core temperature, and the cooling duration was 24 h (Group B).

**Figure 2:**
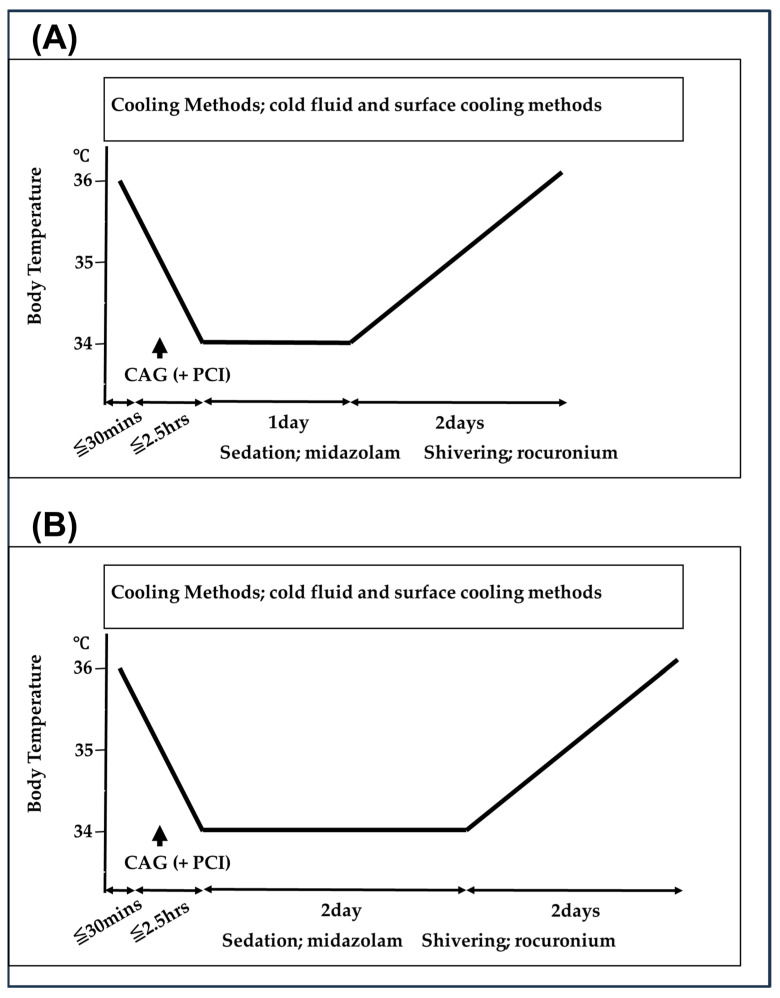
Therapeutic hypothermia protocols from January 2009 to December 2013. CAG indicates coronary angiography; PCI, percutaneous coronary intervention. (**A**) When the time interval from collapse to the return of spontaneous circulation (ROSC) was within 20 min, we performed the 34 °C targeted core temperature, and the cooling duration was 24 h (Group C). (**B**) When the interval was not within 20 min, we performed the 34 °C targeted core temperature, and the cooling duration was 48 h (Group D).

Our therapeutic hypothermia protocol is shown in Figure 1 and Figure 2. For therapeutic hypothermia induction in patients with PCAS, attending physicians managed the patients with lactated Ringer’s solution at 4 °C from emergency department (ED) arrival to intensive care unit (ICU) admission. Urgent coronary angiography (CAG) and percutaneous coronary intervention (PCI) were performed on patients suspected of acute coronary syndrome; as appropriate, during PCI, the treatment of lactated Ringer’s solution at 4 °C was continued. Following ICU admission, we used a surface cooling device using a cooling blanket (Blenketrol II^®^; CSZ Medical, Cincinnati, OH, USA) or an adhesive cooling pad (Arctic Sun^®^, Medivance, Louisville, KY, USA).

We selected the target core temperature and cooling duration for TTM on the basis of the time interval from collapse to ROSC. First, from January 2014 to December 2020, the targeted core temperature was 34 °C or 35 °C, and the cooling duration was 24 h (Figure 1). When the time interval from collapse to ROSC was within 20 min, we performed the 35 °C targeted core temperature (Group A). When the interval was not within 20 min, we performed the 34 °C targeted core temperature (Group B). Conversely, from January 2009 to December 2013, the targeted core temperature was 34 °C, and the cooling duration was 24 or 48 h (Figure 2). When the time interval from collapse to ROSC was within 20 min, we performed the 24 h cooling duration (Group C). When the interval was not within 20 min, we performed the 48 h cooling duration (Group D). The target core temperature was maintained for 24 or 48 h, followed by gradual rewarming (warming by 0.5 °C at 12 h intervals until 36 °C).

### 2.2. Study Participants

Patients with ROSC following in-hospital cardiac arrest (IHCA) or out-of-hospital cardiac arrest (OHCA) and who received therapeutic hypothermia were included. Patients with a tympanic membrane temperature of <30 °C on ED arrival or who had a noncardiac etiology of cardiac arrest were excluded. Participants were divided into the following two groups according to the era of cardiac arrest: Group A + B and Group C + D. Group A + B comprised patients with cardiac arrest from 1 January 2014 to 31 December 2020, and Group C + D encompassed patients with cardiac arrest from 1 January 2009 to 31 December 2013. Additionally, participants were divided into the following four groups according to the time interval from collapse to ROSC: Groups A, B, C, and D.

### 2.3. Role of the Funding Source

This study had no funding source. All authors had full access to all data. The corresponding author had the ultimate responsibility for submitting the study for publication. We prepared the manuscript in accordance with the Strengthening the Reporting of Observational Studies in Epidemiology guidelines.

### 2.4. Endpoints

The primary endpoint included favorable neurological outcomes at 30 days following cardiac arrest, defined as either Cerebral Performance Categories 1 (good performance) or 2 (moderate disability) on a 5-category scale [16]. Unfavorable neurological outcomes were defined as either Cerebral Performance Categories 3 (severe disability), 4 (vegetative state), or 5 (death). Survival (Cerebral Performance Categories 1–4) at 30 days following cardiac arrest was the secondary outcome.

### 2.5. Statistical Analysis

Participants’ baseline characteristics and crude study outcomes were compared using the chi-square and Mann–Whitney U tests for categorical and continuous variables, respectively. Cases with missing data were excluded. Multivariable logistic regression analyses were performed for the independent predictors of the primary endpoint, including study groups as primary exposure variables. Potential confounding factors were selected on the basis of biological plausibility and previous studies and were subsequently included in multivariable logistic regression analyses [7,8,9,17]. Age, sex, cardiac arrest witnessed by somebody or not, presence or absence of bystander CPR, shockable or non-shockable cardiac arrest, IHCA or OHCA, cardiac arrest duration, left ventricular ejection fraction, arterial blood gas pH level, arterial blood gas lactate level, presence or absence of urgent CAG, presence or absence of urgent PCI, performed intra-aortic balloon pumping (IABP) or not, and performed veno-arterial extracorporeal membrane oxygenation (VA-ECMO) or not were the other covariates. Odds ratios (ORs), 95% confidence intervals (CIs), and *p* values were calculated in the multivariable analysis. All hypothesis tests were two-sided, with the significance level was set at <0.05. All statistical analyses were performed using Statistical Package for the Social Sciences (version 25.0 J, SPSS, IBM, Chicago, IL, USA).

## 3. Results

### 3.1. Patient Population and Baseline Characteristics

**Figure 3:**
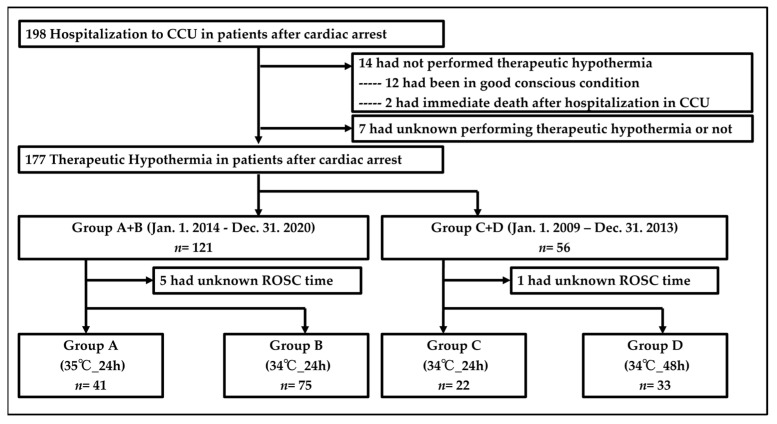
Study profile. ROSC indicates return of spontaneous circulation; CCU, cardiac care unit.

Between 1 January 2009, and 31 December 2020, 198 patients with cardiac arrest were hospitalized in the cardiac care unit (CCU) (Figure 3). A total of 177 patients received therapeutic hypothermia in patients with PCAS. Of them, 121 had cardiac arrest from 1 January 2014 to 31 December 2020 (Group A + B), and 56 had cardiac arrest from 1 January 2009 to 31 December 2013 (Group C + D). Five patients in Group A + B and one patient in Group C + D were missing the duration from collapse to ROSC. Finally, 41, 75, 22, and 33 patients were included in Groups A, B, C, and D, respectively. The baseline characteristics of the two groups showed significant differences in patients with a medical history of diabetes, those who were admitted because of acute coronary syndrome, those who had urgent CAG and PCI, and those who received IABP or VA-ECMO (Table 1).

### 3.2. Outcomes

**Figure 4:**
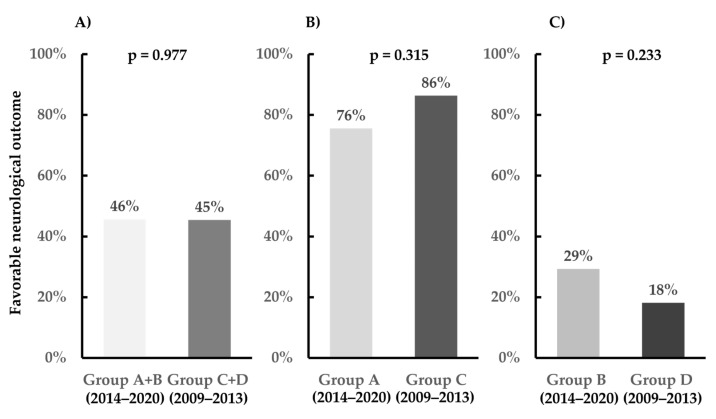
Primary outcomes. (**A**) Whole cohort, (**B**) return of spontaneous circulation (ROSC) within 20 min, and (**C**) ROSC ≥ 21 min.

The outcomes in the entire study population are depicted in Figure 4. The favorable neurological outcome rates at 30 days following cardiac arrest were 45.7% and 45.5% in Groups A + B and C + D, respectively (*p* = 0.977). In the multivariate logistic regression analysis, including the entire study population, the adjusted OR for the neurological intact in Group A + B compared with that in Group C + D was 0.502 (95% CI, 0.133–1.896; *p* = 0.310) (Table 2). In patients with ROSC within 20 min, the favorable neurological outcome rates at 30 days following cardiac arrest were 75.6% and 86.4% in Groups A and C, respectively (*p* = 0.315) (Figure 4). In patients with ROSC ≥21 min, the favorable neurological outcome rates at 30 days following cardiac arrest were 29.3% and 18.2% in Groups B and D, respectively (*p* = 0.233) (Figure 4). The survivals at 30 days following cardiac arrest were 67.8% and 62.5% in Groups A + B and C + D, respectively (*p* = 0.491).

### 3.3. Outcomes in the Subgroups

**Figure 5:**
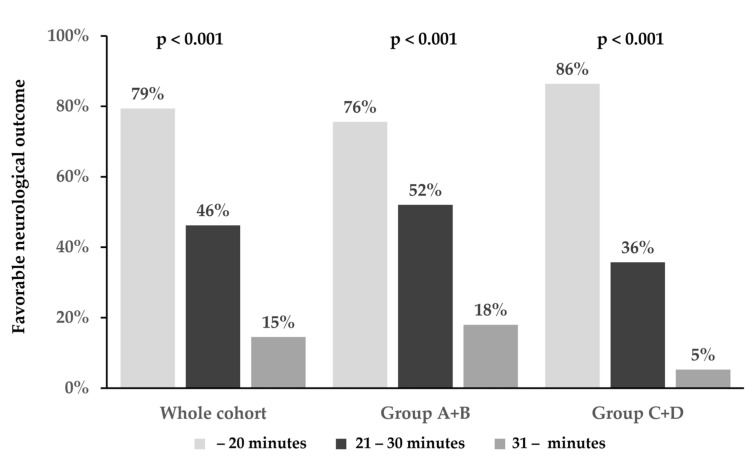
T The favorable 30-day neurological outcomes on the basis of the time interval from collapse to return of spontaneous circulation (within 20 min, 21–30 min, and ≥31 min).

The favorable 30-day neurological outcomes in the subgroups of patients divided into three groups on the basis of the time interval from collapse to ROSC (within 20 min, 21–30 min, and ≥31 min) are shown in Figure 5. In the whole cohort, the favorable 30-day neurological outcome rates following cardiac arrest were 79.4%, 46.2%, and 14.5% in the time intervals of within 20 min, 21–30 min, and ≥31 min, respectively (*p* < 0.001). In Group A + B, the favorable 30-day neurological outcome rates following cardiac arrest were 75.6%, 52.0%, and 18.0% in the time intervals of within 20 min, 21–30 min, and ≥31 min, respectively (*p* < 0.001). In Group C + D, the favorable 30-day neurological outcome rates following cardiac arrest were 86.4%, 35.7%, and 5.3% in the time intervals of within 20 min, 21–30 min, and ≥31 min, respectively (*p* < 0.001).

## 4. Discussion

The results of this study suggested that selecting the optimal target core temperature and cooling duration for TTM on the basis of the time interval from collapse to ROSC can be helpful in patients with PCAS due to cardiac etiology.

We here determined the severity of patients with PCAS by the time interval from collapse to ROSC. Some studies have suggested that measuring initial severity in patients with PCAS can facilitate selecting the optimal TTM strategy [18,19]. Nishikimi et al. showed that risk stratification on the basis of the revised post-cardiac arrest syndrome for therapeutic hypothermia score (rCAST score) was useful for predicting neurologic outcomes in patients with PCAS receiving TTM [19]. The rCAST score was calculated simply by using five clinical factors measured before TTM initiation [18]. The time interval from collapse to ROSC was one factor in the rCAST score. The study reported that dividing the interval into ≤20 or ≥20 min was useful. One study, based on a large database in Japan, showed that, in patients who received therapeutic hypothermia, those with a time to ROSC within 30 min had better neurological outcomes than those with a longer time to ROSC [20]. Moreover, some studies have shown that TTM at 33–34 °C was associated with a significantly higher rate of good neurologic outcomes in moderately severe-to-severe patients with PCAS [18,19,21]. Moderate therapeutic hypothermia improves neurological outcomes in patients with severe ischemia–reperfusion brain injury following cardiac arrest [22,23].

In our study, we divided the time interval from collapse to ROSC into ≤20 or ≥21 min. The severity of patients with PCAS with a time interval of ≥21 min could be considered moderate and severe. In these patients, treating the TTM at 34 °C may be necessary. Furthermore, when the participants were divided into three groups according to the time intervals of ≤20 min, 21–30 min, and ≥31 min, favorable neurological outcomes were significantly different among the three groups (Figure 5). This result suggested that patients with PCAS with the 21–30 min time interval had moderate severity. Further study is needed regarding the optimal target core temperature in patients with the time interval of 21–30 min.

A 24 h cooling duration may be optimal in patients with PCAS with any severity but not 48 h. Kirkegaard et al. showed that the 48 h cooling duration at 33 °C had a significantly higher proportion of patients with one or more adverse events than the 24 h cooling duration at 33 °C [9]. However, compared with TTM for 24 h, TTM for 48 h did not significantly improve neurologic outcomes. Therapeutic hypothermia causes several adverse events, including arrhythmias, bleeding, skin complications, pneumonia, and sepsis [7,10,24]. The TTM2 trial showed that arrhythmias resulting in hemodynamic compromise were significantly common in patients who maintained 33 °C as the target core temperature [7]. In our study, the incidence of major adverse events and the neurological intact did not significantly differ between each group. The results of our study suggested that a cooling duration can be optimal for 24 h.

This study had several limitations. First, it was neither a randomized controlled trial nor a multicenter trial. Second, all data were collected from medical records. Although no significant difference in the incidence of major adverse events was observed between the groups, the incidence of minor adverse events, which were not documented in the medical records, was unknown. In addition, no cases of TTM were interrupted due to complications of therapeutic hypothermia in medical records. Third, we did not discuss the period of rewarming in this study. We performed rewarming at a constant pace (warming by 0.5 °C at 12 h intervals until 36 °C). However, the rewarming period was 1 day in Group A and 2 days in Groups B, C, and D. Further study is needed regarding the optimal rewarming period. Fourth, this study included hospitalized cardiac arrest patients in CCU due to cardiac causes, which may not reflect the total number of patients with PCAS treated. Finally, although the guidelines for the international consensus on CPR and Science with Treatment Recommendations were updated during the study period [2,25,26,27], the guidelines update may not have influenced this study. Although a nationwide population-based registry study in Japan reported that bystander CPR implementation increased annually by the guidelines update [28], no significant difference in bystander CPR was noted between Groups A + B and C + D. Conversely, Group A + B had a significantly higher number of implementations of post-cardiac arrest care, including CAG, PCI, IABP, and VA-ECMO, than Group C + D according to the guidelines update. However, the multivariate logistic regression analysis showed that post-cardiac arrest care was not an independent predictor of neurological intact.

## 5. Conclusions

Selecting the optimal target core temperature and cooling duration for TTM on the basis of the time interval from collapse to ROSC may be helpful in patients with PCAS due to cardiac etiology.

## Figures and Tables

**Table 1:** Baseline characteristics.

Characteristic	Group A + B	Group C + D	*p* Value
Age—yr	63 ± 15	64 ± 12	0.802
Male sex—no. (%)	100 (82.6)	43 (76.8)	0.357
Medical history Hypertension—no (%)	68 (56.2)	31 (55.4)	0.917
Medical history Diabetes—no (%)	45 (37.2)	12 (21.8)	0.043
Medical history Myocardial infarction—no (%)	31 (25.6)	14 (25.0)	0.930
Medical history Heart failure—no (%)	19 (33.9)	30 (24.8)	0.207
Witness Cardiac Arrest—no (%)	78 (60.9)	38 (59.4)	0.835
Bystander CPR ^1^—no (%)	68 (53.1)	26 (40.6)	0.102
Shockable rhythm—no (%)	94 (74.0)	44 (68.8)	0.443
Location Place of residence—no (%)	55 (43.0)	31 (48.4)	0.473
Location Public place—no (%)	58 (45.3)	27 (42.2)	0.681
Location Hospital—no (%)	16 (12.5)	5 (7.8)	0.327
Duration of cardiac arrest—min	31 ± 20	28 ± 20	0.188
Ejection fraction—%	46 ± 17	50 ± 18	0.179
Blood gas analysis Ph ^2^	7.09 ± 0.21	7.12 ± 0.23	0.300
Blood gas analysis Lactate ^3^—mmol/L	9.61 ± 3.68	11.28 ± 0.14	0.317
Cause of Cardiac arrest			0.044
Acute Coronary Syndorome—no (%)	55 (43.0)	16 (25.0)	
Congestive Heart Failure—no (%)	12 (9.4)	11 (17.2)	
Old Myocardial Infarction—no (%)	19 (14.8)	12 (18.8)	
Arrhythmia—no (%)	9 (7.0)	10 (15.6)	
Cardiomyopathy—no (%)	12 (9.4)	5 (7.8)	
Pulmonary Embolism—no (%)	2 (1.6)	3 (4.7)	
Vasospastic Angina—no (%)	10 (7.8)	1 (1.6)	
Others—no (%)	9 (7.0)	6 (9.4)	
Urgent coronary angiography—no (%)	104 (81.3)	30 (46.9)	<0.001
Urgent PCI ^4^—no (%)	52 (40.6)	11 (17.2)	0.001
IABP ^5^—no (%)	91 (71.1)	19 (30.2)	<0.001
VA-ECMO ^6^—no (%)	17 (13.3)	2 (3.1)	0.026

^1^ CPR indicates cardiopulmonary resuscitation. ^2^ Total number of patients; 158 in Ph, and ^3^ 153 in lactate. ^4^ PCI indicates percutaneous coronary intervention; ^5^ IABP, intra-aortic balloon pumping; ^6^ VA-ECMO, veno-arterial extracorporeal membrane oxygenation.

**Table 2:** The adjusted OR for the neurological intact.

Variable	Adjusted OR	95% CI	*p* Value
Age	0.948	0.906–0.992	0.022
Male sex (Reference Female)	1.432	0.385–5.327	0.592
Witness Cardiac Arrest	0.586	0.165–2.076	0.407
Bystander CPR ^1^	0.482	0.155–1.500	0.208
Shockable rhythm (Reference Non-shockable rhythm)	7.128	1.744–29.13	0.006
Location Out of Hospital (Reference in Hospital)	28.27	2.103–379.9	0.012
Duration of cardiac arrest	0.882	0.832–0.935	<0.001
Ejection fraction	1.013	0.981–1.046	0.430
Ph	185.2	4.467–7681.3	<0.001
Lactate	1.202	0.984–1.468	0.071
Urgent coronary angiography	4.392	0.440–43.89	0.208
Urgent PCI ^2^	1.582	0.423–5.911	0.495
IABP ^3^	0.254	0.031–2.050	0.198
VA-ECMO ^4^	2.014	0.111–36.55	0.636
Group A + B (Reference Group C + D)	0.502	0.133–1.896	0.310

^1^ CPR indicates cardiopulmonary resuscitation; ^2^ PCI, percutaneous coronary intervention; ^3^ IABP, intra-aortic balloon pumping; ^4^ VA-ECMO, veno-arterial extracorporeal membrane oxygenation.

## Data Availability

The raw data supporting the conclusions of this article will be made available by the authors upon request.
